# Paediatric neuromuscular scoliosis and post-operative blood pressure targets: a retrospective analysis

**DOI:** 10.1007/s43390-025-01200-1

**Published:** 2025-10-24

**Authors:** Grace Pulling, Lionel D. Rayward, Anthony Slater, Maree T. Izatt, Adam F. Parr, Simon C. Gatehouse, Robert D. Labrom, Geoffrey N. Askin, J. Paige Little

**Affiliations:** 1 Biomechanics and Spine Research Group, School of Mechanical, Medical and Process Engineering, Queensland University of Technology (QUT), South Brisbane, Australia; 2 Centre for Biomedical Technologies, Faculty of Engineering, Queensland University of Technology (QUT), Brisbane, Australia; 3 Department of Orthopaedics, Children’s Health Queensland Hospital and Health Service, South Brisbane, Australia; 4 Department of Paediatric Intensive Care Medicine, Children’s Health Queensland Hospital and Health Service, South Brisbane, Australia

**Keywords:** Blood pressure, Spine deformity, Delayed spinal cord injury, Mean arterial pressure, Neuromuscular scoliosis, Paediatrics

## Abstract

**Purpose:**

This study investigates blood pressure variations and clinical outcomes in paediatric neuromuscular scoliosis patients after deformity correction surgery to mitigate the risk of ischaemic spinal cord injury (SCI). Hypotension is proposed as an aetiological mechanism for delayed SCI, but there is limited evidence regarding the frequency, severity, duration, and clinical effect of hypotension exposure in the immediate post-operative period.

**Methods:**

This is a retrospective review of 94 patients with cerebral palsy or CP-like conditions, who underwent posterior spinal instrumentation at Queensland Children's Hospital. Post-operative mean arterial pressure (MAP) variations and associations with tissue perfusion markers were analysed. Hypotension was described using area under threshold (AUT) and time under threshold (TUT) for MAP thresholds of 40–80 mmHg.

**Results:**

14.9% of patients experienced an episode of hypotension under 60 mmHg lasting ≥ 60 min, compared to 62.4% at 70 mmHg. At the 60 mmHg threshold, mean TUT was 9.2%, 95% CI [6.55, 11.8], compared to 40.9%, 95% CI [35.1, 46.6] at 70 mmHg. Logistic regression revealed increased hypotension exposure under thresholds of 60 and 65 mmHg over a 60-min period was associated with increased risk of hyperlactataemia (AUT, 60 mmHg threshold β = 2.85, OR 17, *p* = 0.03, 95% CI [0.22—5.48]. Increased AUT exposure was associated with low urine output at thresholds ≥ 60 mmHg over 30 min. Haemoglobin ≤ 80 g/L was associated with elevated lactate.

**Conclusion:**

Periods of sustained hypotension were common and usually without neurologic sequalae; however, a MAP below 60 and 65 mmHg sustained for 60 min was associated with surrogate markers of global tissue hypoperfusion.

**Supplementary Information:**

The online version contains supplementary material available at 10.1007/s43390-025-01200-1.

## Introduction

Paediatric spinal deformity surgery is a highly specialised area of orthopaedics, requiring a synergistic approach between surgical, anaesthetic, and intensive care teams [1, 2]. Neuromuscular scoliosis (NMS) patients comprise a large subset of the cohort requiring surgical intervention [3, 4]. Post-operative neurological monitoring is crucial but often challenging in patients with pre-existing deficits, who are unable to understand and follow commands, and unable to report changes readily. While permanent neurological deficits peri-operatively are rare, they remain a significant concern for patients and families, with the overall neurological deficit rate reported between 0.71 and 0.94% [5]. The incidence of delayed spinal cord injury (DSCI) appears to be low; however, it is relatively unknown [6, 7]. Rutges et al. suggest a DSCI incidence of 0.5% in NMS, with neurological deficits first diagnosed mean 16 h (range 2.5–40) after surgery [8].

Hypotension is recognised as a potential precipitant of neurological injury, particularly in the immediate post-operative period when spinal cord autoregulation may be impaired by increased physiological and mechanical stress [7, 9–12]. Several case studies report paediatric DSCI [13–18], and some present the compounding effect of anaemia on episodes of post-operative hypotension exposure, exacerbating the risk of neurological complications. Currently, there is no consensus on post-operative mean arterial pressure (MAP) targets for the NMS population. High-quality evidence comparing MAP targets in critically ill children is limited at present [19–21], with at least one trial underway exploring permissive hypotension [22]. Practitioners often draw from adapted guidelines from traumatic spinal cord injury (SCI) cohorts despite limited evidence and applicability to the peri-operative NMS cohort [23–27].

Vasoactive medications are frequently used peri-operatively and following SCI to maintain adequate blood pressure (BP). However, they carry risk, especially when administered through peripheral lines. Central venous access devices (CVADs) are often sought to facilitate vasopressor administration but also have associated complications [28–30]. Lactate and urine output are utilised in the intensive care setting as proxy markers of end-organ perfusion [31–40].

This study seeks to define hypotension thresholds and analyse risk factors for impaired tissue perfusion in NMS patients following spinal deformity stabilisation and correction surgery. By defining normal BP variations for this vulnerable population, this study will assist in identifying patients who deviate from the normal post-operative course. This may aid in informing future approaches to clinical management for this patient group, mitigating the risk of ischaemic spinal cord injury while avoiding unnecessary BP interventions.

## Materials and methods

### Study design

A single-centre, retrospective study was carried out with patient health data from the Queensland Children’s Hospital (QCH), Brisbane, Australia, including orthopaedic surgical data and paediatric intensive care unit (PICU) data. The population included a convenience sample. A convenience sample was used in this study, meaning all eligible patients meeting inclusion criteria within the specified study period at our institution were included, rather than a randomly selected or consecutively recruited cohort of 94 paediatric NMS patients (aged 6–18 years) having undergone posterior spinal instrumentation (101 cases) at QCH from January 2015 to December 2023 inclusive, who were admitted to PICU post-operatively.

During the study period (2015 – 2023), 285 neuromuscular surgery cases were identified in the hospital’s orthopaedic spinal database. Patients were screened sequentially according to eligibility: (1) meeting neuromuscular diagnosis type and relevant demographic criteria, (2) type of surgical procedure, and (3) GMFCS status. Following this, 102 patients remained who were all admitted to PICU post-operatively; 1 patient was excluded due to missing invasive blood pressure monitoring, resulting in 101 included cases. These details are illustrated in the CONSORT-style flow diagram provided in the Author’s Response to Reviewers’ Comments document.

This study was conducted in full conformance with principles of the Declaration of Helsinki and the National Health and Medical Research Council National Statement on Ethical Conduct in Human Research, with both health service and university Human Research Ethics Committee (HREC) approvals [41, 42]. As this was a retrospective analysis of de-identified cohort data, the requirement for individual patient consent was waived by the lead HREC.

#### Inclusion criteria

Included patients were those aged ≤ 18 years having a background of progressive NMS and a diagnosis of Cerebral Palsy (CP) or CP-Like condition, with a Gross Motor Function Classification System (GMFCS) levels III–IV, admitted to PICU post-operatively with invasive arterial monitoring [43, 44]. Included procedures were posterior spinal instrumented fusion (PSIF), insertion of growing rods, and significant revision procedures at QCH between 2015 and 2023 inclusive.

#### Exclusion criteria

Excluded patients were those functioning at a GMFCS level I-II, those with muscular dystrophy, neurofibromatosis, pre-existing SCI, connective tissue disease and idiopathic, congenital or syndromic scoliosis. Typically, these patients can communicate well, permitting reliable neurological assessment and diminishing the need for surrogate markers of tissue perfusion. Exclusion of these conditions also allowed for examination of a cohort with homogenous physiological status and co-morbidity burden. Excluded procedures were removal of metalware, lengthening of growing rods, surgical management of wound infection, and revision procedure whereby there is limited instrumentation. Patients admitted directly to the ward post-operatively were excluded.

### Data Collection

PICU data were extracted from the Clinical Information System (CIS) (MetaVision ICU™). Medical documentation for the remainder of each patient's admission at QCH was manually reviewed via the electronic medical record and the CIS by the primary investigator. All patient data were de-identified for analysis. Files were stored electronically on a secure, regularly backed-up access server.

### Outcomes

**Fig. 1:**
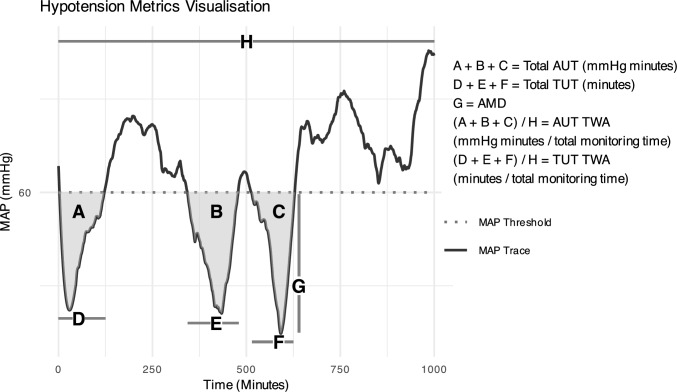
Hypotension exposure metrics visualisation. *MAP* mean arterial pressure, *AUT* area under threshold, *TUT* time under threshold, *AMD* absolute maximum decrease (below specified MAP threshold), *TWA* time-weighted average (also referred to as "normalised", total monitoring time = duration of invasively monitored blood pressure ≤ 24 h)

The primary outcomes included raw BP measurements and biomarkers utilised to approximate tissue perfusion in PICU (Supplementary Table 1 and Fig. 1). These were collated and analysed for the initial 24 h of the admission to PICU. CIS data included: minutely arterial line BP monitoring (until 24 h or cessation of monitoring), four-hourly non-invasive BP readings, heart rate, temperature, biochemical markers (lactate, creatinine, haemoglobin, haematocrit), and hourly urine output (UO). Acute kidney injury (AKI) was defined according to Kidney Disease for Improving Global Outcomes AKI criteria, with serum creatinine monitored for up to 72 h following PICU admission. Pre-operative creatinine, haemoglobin, and blood pressure measurements were also collected for comparison with post-operative data.

The secondary outcomes included clinical interventions (fluid therapy, vasoactive drug therapy, blood product administration, central line placement), necessity for advanced imaging, adverse outcomes such as neurological deficit, AKI, major cardiac and respiratory complications. Patient demographics, major scoliosis Cobb angle, operative data, and length of stay (LOS) were also recorded.

### Defining Hypotension Exposure

MAP area under threshold (AUT in mmHg*minute) and time under threshold (TUT in minutes) were the primary metrics used (Fig. 1) [45]. We analysed variations including total sub-threshold exposure, exposure in the preceding 30, 60, and 120 min before specific time points (i.e. lactate reading), and normalised AUT and TUT (AUT, TUT divided by total monitoring time). Additional exposure definitions included hypotensive episodes categorised by duration and severity and absolute maximum decrease (AMD). Higher thresholds were explored given the varied recommendations in the literature to target 80 mmHg or above, following paediatric SCI or intra-operative neuromonitoring (IONM) change; however, evidence is scant [23, 24, 27].

### Statistical analysis

Statistical analyses were performed using R Statistical Software (v4.4.1; R Core Team 2024) 4.4.1[46]. Raw invasive BP data underwent artefact filtering using a previously validated algorithm [47, 48].

Hypotension exposure was quantified for individual subjects using various threshold definitions (Fig. 1). Descriptive statistics were calculated for all outcomes.

Logistic regression was performed using “glmer” function via R package lme4 (v. 1.1.35.5), to explore relationships between variables (lactate, UO, AKI, Hb) and BP [49]. Linear regression (using the “lm” function via R package lme4) was used to assess the differences between blood pressure readings at varied time blocks (4-hourly). Varied analyses of continuous BP against dichotomous and continuous surrogate biomarkers of tissue perfusion were undertaken (Supplementary Table 1, Fig. 1). All datapoints were analysed for the entire cohort. Following this, multiple analyses were performed with different filtering criteria for vasopressor use: exclusion of patients who received vasopressor therapy, exclusion of data from the time of vasopressor administration onwards, and exclusions of data 30, 60, or 120 min following vasopressor administration. This comprehensive approach allowed for a thorough examination of relationships between BP and markers of perfusion, accounting for the potential confounding effects of vasopressor use, such as lactate rise associated with the commencement of catecholamine vasopressors. Logistic regression modelling describes the estimate, which represents the change in odds of risk of a negative outcome (i.e. elevated lactate, low UO) for each additional unit of mmHg*hour (AUT) and hour (TUT) spent under a specific MAP threshold.

For all statistical tests, a nominal p-value < 0.05 was considered statistically significant. To explore potential subgroups within the cohort, k-means cluster analysis was performed. Additionally, outlier detection was conducted to identify and assess the impact of extreme values.

## Results

### Demographics, admission, and surgical data

This study evaluated 94 patients (101 surgical cases). The cohort was 53.5% female with a mean age of 13.2 years (range 6.9–18.6) and mean weight of 35.5 kg (range 15.4–81.5). CP was the primary diagnosis in 81.9% of patients. Nearly all cases (97%) were classified as American Society of Anesthesiologists (ASA) physical status 3. Salient co-morbidities are listed in Table 1. The mean pre-operative major scoliosis Cobb angle was 82.7° (range 50.0–120.0, *n* = 67 (available at time of analysis)). The median PICU length of stay (LOS) was 24.9 h (range 19.9–765.8), while the median hospital LOS was 8.3 days (range 3.5–95.3); 84 of 101 procedures were primary PSIF. Most cases involved proximal instrumentation at T2 or T3 and extended distally to the pelvis. One patient underwent a Smith-Petersen osteotomy, another had a single-level pedicle subtraction osteotomy, and a third had a Ponte osteotomy. In addition, 11 cases involved central decompression to facilitate sublaminar wire placement. All patients underwent partial facetectomies prior to bone grafting. Estimated blood loss (EBL) was not analysed due to inconsistent data availability. Epidurals were placed surgically prior to closure in 87 cases (Table 1).

**Table 1:** Demographics, co-morbidities, and surgical data

Demographics and Co-morbidities	Count (% Cohort)	Surgical Characteristics	Count
CP	77 (81.9%)	Primary PSIF	84
CP-Like	17 (18.1%)	Insertion of Growing Rods	8
GMFCS: V	55 (58.5%)	Exchange of Growing Rods	1
GMFCS: IV	32 (34.0%)	Revision of Bipolar Fixation to PSIF	2
GMFCS: III	7 (7.4%)	Conversion of Growing Rods to PSIF	2
Primary Neuromuscular Condition: CP	77 (81.9%)	Revision of Proximal PSIF Construct	2
Primary Neuromuscular Condition: Rett Syndrome	12 (12.8%)	Revision to Proximal and Distal Construct	1
ASA: 3	98 (97.0%)	Revision of Growing Rods	1
ASA: 4	2 (2.0%)	Proximal Instrumented Level: T2	38
ASA: 2	1 (1.0%)	Proximal Instrumented Level: T3	46
Movement Classification: Spastic quadriplegia	38 (48.7%)	Proximal Instrumented Level: T4	17
Previous Orthopaedic Surgery (non-spinal)	42 (44.7%)	Distal Instrumented Level: L2	1
Visual Impairment	30 (31.9%)	Distal Instrumented Level: L3	5
Gastroesophageal Reflux Disease	19 (20.2%)	Distal Instrumented Level: L4	7
Gastrostomy	63 (67.0%)	Distal Instrumented Level: L5	7
Genetic Mutation	13 (13.8%)	Distal Instrumented Level: Pelvis	81
Intrathecal Baclofen Pump	12 (12.8%)	Epidurals Placed	87
Ventriculoperitoneal Shunt	6 (6.4%)		
Deep Brain Stimulator	1 (1.1%)		

### Hypotension exposure

#### Episodes by duration and severity thresholds

**Fig. 2:**
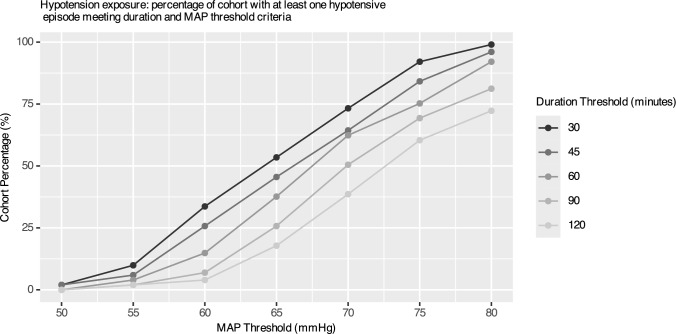
Percentage of cohort with at least one hypotensive episode meeting duration and MAP threshold criteria. MAP thresholds 50–80 mmHg are displayed here

When defining hypotension exposure by episodes of varying durations at varying MAP thresholds, we observed a significant increase in episode frequency across the cohort as the threshold transitioned from 55 to 65 mmHg (Table 2, Fig. 2). For shorter episodes (30 and 60 min), there was a significant increase in episode frequency across the cohort when transitioning from 55 to 60 mg, i.e. at the 30-min duration, there was a relative increase of 450% (from 14 to 77 episodes). This transition point moved to the 60–65 range for the longer durations (90 and 120 min).

**Table 2:** Hypotension exposure: episode counts and cohort percentage meeting duration (30, 60, 90, and 120 min thresholds displayed here) and MAP threshold criteria

	MAP Threshold	Duration	Episode Count	Relative Change (%) of Episodes	Individual Patient Count	Episode Mean	Episode SD
	50	30	3	NA	2	0.03	0.22
	55	30	14	367	10	0.13	0.42
	60	30	77	450	34	0.76	1.41
	65	30	189	145	54	1.87	2.48
	70	30	344	82	74	3.36	3.08
	75	30	460	33.7	93	4.51	2.89
	80	30	487	5.87	100	4.77	2.79
	50	60	0	NA	0	0	0
	55	60	5	NA	4	0.05	0.26
	60	60	21	320	15	0.21	0.55
	65	60	84	300	38	0.83	1.39
	70	60	181	115	63	1.77	1.99
	75	60	248	37	76	2.45	1.96
	80	60	280	12.9	93	2.75	1.76
	50	90	0	NA	0	0	0
	55	90	2	NA	2	0.02	0.14
	60	90	9	350	7	0.09	0.38
	65	90	42	367	26	0.42	0.8
	70	90	109	160	51	1.07	1.32
	75	90	171	56.9	70	1.68	1.5
	80	90	201	17.5	82	1.95	1.49
	50	120	0	NA	0	0	0
	55	120	2	NA	2	0.02	0.14
	60	120	5	150	4	0.05	0.26
	65	120	27	440	18	0.27	0.65
	70	120	69	156	39	0.65	1.01
	75	120	121	75.4	61	1.18	1.21
	80	120	148	22.3	73	1.44	1.29

AUT/TUT	MAP Threshold	Whole Cohort Mean	Whole Cohort Median	Relative Change (%) of Median	95% CI	Vasopressor Cohort Mean	Vasopressor Cohort Median	95% CI
AUT	50	0.02	0.00	NA	0.01–0.04	0.02	0	0.01–0.03
AUT	55	0.11	0.01	NA	0.06–0.16	0.14	0.06	0.06–0.21
AUT	60	0.42	0.08	700	0.27–0.56	0.58	0.39	0.33–0.84
AUT	65	1.24	0.5	525	0.92–1.56	1.75	1.45	1.1–2.4
AUT	70	2.9	2.09	318	2.33–3.47	3.91	3.82	2.69–5.12
AUT	75	5.49	4.91	135	4.65–6.32	6.95	7.34	5.17–8.73
AUT	80	8.86	8.61	75.4	7.8–9.92	10.7	11.2	8.53–12.9
TUT	50	0.74	0.00	NA	0.31–1.16	0.83	0.11	0.23–1.42
TUT	55	2.74	0.16	NA	1.66–3.81	3.86	2.01	1.87–5.84
TUT	60	9.16	2.41	1410	6.55–11.8	13.6	9.48	8.15–19.1
TUT	65	22.40	14.9	517	17.8–27.0	31.1	30.2	20.9–41.3
TUT	70	40.90	39.8	168	35.1–46.6	50.9	57.3	38.3–63.4
TUT	75	58.50	63.40	59.3	52.8- 64.2	67.2	74.5	56.3–78.1
TUT	80	72.90	80.10	26.4	68.2- 77.7	81.0	86.7	73.1–88.9

Relative change represents the relative increase in the number of hypotensive episodes observed across the cohort when increasing the blood pressure threshold from the value of the preceding 5 mmHg bracket. Episode number means and standard deviations (SD) are also calculated. Normalised AUT (mmHg*min/min) and normalised TUT (%) descriptive statistics (whole cohort compared to vasopressor cohort) are also shown alongside relative percentage change

#### Area under the threshold

**Fig. 3:**
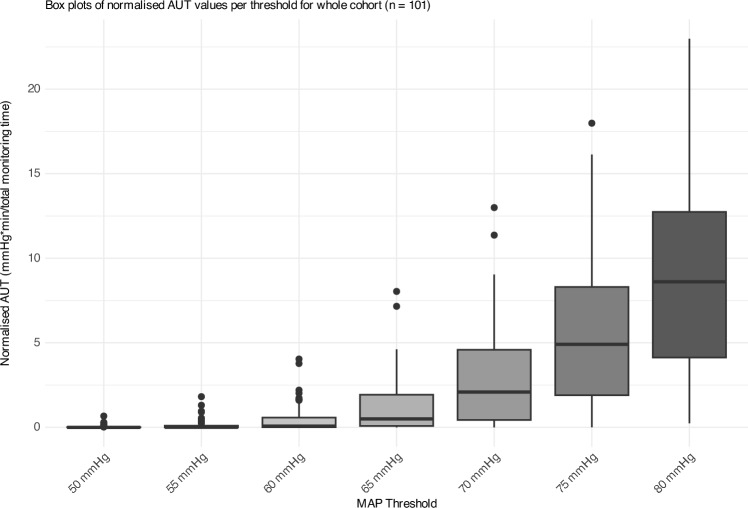
Box plots for normalised AUT across varying MAP thresholds for the whole cohort (*n* = 101)

Normalised AUT (time-weighted average (mmHg*min/min)) analysis reveals distinct patterns across different BP ranges (Table 2, Fig. 3). More than half of patients never experienced exposure under 55 mmHg (median 0.01 mmHg*min/min at 55 mmHg). A significant transition occurs between 55 and 60 mmHg, with median normalised AUT increasing from 0.01 to 0.08 mmHg*min/min (relative change 700%). Percentage change from thresholds 60–65 mmHg was also high at 525%.

#### Time under the threshold

Few patients maintain MAP below the lower thresholds (40, 45, 50 and 55 mmHg), with medians consistently close to zero at the 50 mmHg threshold (Table 2). Significant increases in both mean and median values are observed at thresholds 55 and 60 mmHg, with patients spending a median 0.16% of the monitoring period below 55 mmHg, increasing to 2.41% at 60 mmHg. AUT results are also reflected in the TUT results for the comparison of thresholds 60 and 65 mmHg. Most patients spend a significant portion of time below the higher thresholds, with the median reaching 80.10% at 80 mmHg. Patients who have received vasopressor intervention spent 293% more time under a 60 mmHg threshold than the remainder of the cohort (medians of 9.48% and 2.41%, respectively).

#### Baseline blood pressure

Post-operative invasive BP readings were analysed and compared with the nearest non-invasive pre-operative reading (baseline). This baseline was available for 86 surgical cases and was taken from 5.3 to 40 h prior to PICU admission. Post-operative readings were grouped into six 4-h time blocks to analyse trends over the 24-h period using linear modelling. Post-operative invasive MAP was significantly lower than pre-operative baseline values across all time blocks in the first 24 h after surgery. The largest reduction in MAP from baseline (mean 80.1 mmHg) occurred between 8 and 12 h (mean 71.6 mmHg) post-operatively, with a mean decrease of 8.5 mmHg (p < 0.001). MAP began to recover after 16 h, with a significant increase from 8 to 12 h and, however, did not fully return to baseline values within the studied 24-h period. Of note, non-invasive pre-operative baseline BP readings in the paediatric population are often confounded by factors such as anxiety and sedating medications.

### Markers of tissue perfusion

#### Lactate

The effects of hypotension as defined by different thresholds and pre-measurement periods were modelled against lactate (as a dichotomous variable ≥ 2 mmol/L, Supplementary Table 2). Multivariable logistic regression analysis revealed that after accounting for the effects of age as a covariate, increasing AUT and TUT increased the risk of elevated lactate at thresholds 60, 65, 70, and 75 mmHg, with the strength and significance of the association varying by threshold and pre-measurement period (Table 3, Supplementary Table 3). Models for thresholds < 60 mmHg for the 60-min pre-measurement period were not statistically significant. The trend suggested that monitoring AUT, for thresholds of 60 and 65 mmHg over the 60- and 120-min monitoring periods, was associated with elevated lactate levels (60 mmHg threshold for 60-min pre-measurement period β = 2.85, OR 17.35, *p* = 0.03, 95% CI [0.22—5.48], Table 3).

**Table 3:** Multivariable logistic regression analysis of AUT effect (continuous) on lactate (dichotomous), with age controlled for as a covariate

Pre-Measurement Period	MAP Threshold	AUT β Estimate	AUT 95% CI	AUT SE	AUT OR	AUT *p*
30	50	8.65	-12.28—29.59	10.68	5734	0.42
30	55	2.77	-3.97—9.51	3.44	16	0.42
30	60	2.49	-0.78—5.76	1.67	12	0.14
30	65	1.77	-0.00—3.53	0.90	5.84	0.05
30	70	1.06	-0.03—2.16	0.56	2.90	0.06
30	75	0.72	-0.06—1.49	0.40	2.05	0.07
30	80	0.46	-0.16—1.08	0.31	1.58	0.14
60	50	10.88	-11.84—33.60	11.59	53 092	0.35
60	55	3.99	-1.98—9.96	3.05	54	0.19
60	60	2.85	0.22—5.48	1.34	17	0.03
60	65	1.58	0.36—2.81	0.62	4.88	0.01
60	70	0.81	0.13—1.49	0.35	2.25	0.02
60	75	0.49	0.03—0.95	0.24	1.63	0.04
120	50	5.63	-6.76—18.03	6.32	279	0.37
120	55	2.76	-1.06—6.58	1.95	16	0.16
120	60	1.62	0.14—3.10	0.75	5.05	0.03
120	65	0.71	0.08—1.34	0.32	2.04	0.03

AUT values rescaled to mmHg*hours (from mmHg*mins). ORs indicate odds ratios; SE indicates standard error; CI indicates confidence intervals

#### Urine output

Logistic regression modelling of the effects of AUT and TUT on UO (as a dichotomous variable ≤ 0.5 ml/kg/hr) under different thresholds and pre-measurement periods found that increased AUT was significantly associated with low urine output for thresholds ≥ 60 mmHg (30-min pre-measurement period) and 65 mmHg (60 min pre-measurement period), i.e. AUT at the 60 mmHg threshold for the 30-min pre-measurement period β = 0.20, OR 1.22, p = 0.01, CI [0.05—0.36]. For both AUT and TUT analyses (Supplementary Tables 4 and 5, respectively), lower MAP thresholds (40–55 mmHg) generally showed non-significant associations, often with negative or weak relationships. The strength of the relationship typically plateaued at thresholds ≥ 70 mmHg and weakened as the pre-measurement period lengthened.

#### Anaemia

Post-operative Hb descriptive statistics are displayed in Supplementary Table 2. Mean pre-operative haemoglobin was 139.9 g/L (SD 15.34, range 106 –178, n = 100) while mean post-operative haemoglobin was 99.60 g/L (SD 18.32, range 40 –159, n = 586). Multivariable logistic regression modelling explored the effects of haemoglobin and blood pressure on lactate levels, with age as a covariate. (AUT and TUT analysis is displayed in Table 4 and Supplementary Table 6, respectively.) Haemoglobin was categorised based on the minimum value during the pre-measurement period: ≤ 80, 80–100, and > 100 g/L. When compared with haemoglobin > 100 g/L, haemoglobin values ≤ 80 g/L were significantly associated with increased risk of elevated lactate for some MAP thresholds for the 30-min pre-measurement period (AUT analysis Hb ≤ 80 g/L, 70 mmHg threshold β = 1.78, OR 5.96, p = 0.04, 95% CI [0.06—3.51]; TUT analysis Hb ≤ 80 g/L, 65 mmHg threshold β = 1.73, OR 5.63, p = 0.04, 95% CI [0.03—3.43]).

**Table 4:** Multivariable logistic regression analysis of haemoglobin (categorical ≤ 80 g/L against > 100 g/L) with AUT (continuous) on lactate (dichotomous) and age controlled for as a covariate

Pre-Measurement Period	MAP Threshold	Hb ≤ 80 g/L β Estimate	Hb ≤ 80 g/L 95% CI	Hb ≤ 80 g/L SE	Hb ≤ 80 g/L OR	Hb ≤ 80 g/L *p*
30	50	1.38	-0.28—3.03	0.84	3.97	0.10
30	55	1.43	-0.24—3.10	0.85	4.17	0.09
30	60	1.52	-0.16—3.20	0.86	4.57	0.08
30	65	1.69	-0.01—3.39	0.87	5.42	0.05
30	70	1.78	0.06—3.51	0.88	5.96	0.04
30	75	1.74	0.03—3.45	0.87	5.70	0.046
30	80	1.81	0.08—3.53	0.88	6.09	0.04
60	50	1.41	-0.38—3.21	0.92	4.11	0.12
60	60	1.48	-0.29—3.24	0.90	4.39	0.10
60	65	1.56	-0.21—3.32	0.90	4.74	0.08
60	70	1.56	-0.19—3.31	0.89	4.75	0.08
120	50	1.81	-0.63—4.24	1.24	6.09	0.15
120	55	1.75	-0.59—4.10	1.20	5.76	0.14
120	60	1.68	-0.58—3.94	1.15	5.35	0.15
120	65	1.66	-0.55—3.87	1.13	5.24	0.14

AUT values rescaled to mmHg*hours (from mmHg*mins). ORs indicate odds ratios; SE indicates standard error; CI indicates confidence intervals

### Clinical interventions

#### Vasoactive drug therapy

A total of 22 patients required vasopressor intervention, with 13 commenced within 4 h of PICU admission after surgery. Mean therapy duration was 129.36 min. Medications included metaraminol (*n* = 17), noradrenaline (*n* = 4), and adrenaline (*n* = 4). For a 60 mmHg threshold, median TUT decreased from 30.65% pre-vasopressor to 13.66% during intervention. Normalised AUT/TUT illustrated increased hypotension exposure compared to the whole cohort (Table 2).

#### Central line access

In total, 15 patients had a CVAD (4 placed in PICU), including 5 of the 22 patients who received vasopressor intervention.

### Adverse outcomes

#### Neurological

Two patients sustained permanent neurological deficits. A 13-year-old female with Rett syndrome developed upper and lower limb weakness within 24 h post-operatively. Immediate post-operative neurological examination elicited baseline neurology and IONM did not demonstrate significant reductions or alerts, with baseline responses maintained. Following onset of new neurological deficits, MRI demonstrated central cervical spinal cord T2 hyper-intensity, suggesting myelitis or early cord ischaemia, consistent with C5/6 SCI. She spent significantly more time below a 60 mmHg threshold (normalised AUT 4.04 mmHg*min/min), falling above the upper 95th percentile of 0.56 mmHg*min/min for the cohort, compounded by anaemia (Hb 69 g/L). She sustained low urine output for 4 consecutive hours and 5 elevated lactate readings.

An 18-year-old male with CP and complex hypertonic movement disorder developed new upper and lower limb weakness following a cardiac arrest on post-operative Day 0, likely due to autonomic dysfunction, requiring 2 min of CPR and adrenaline followed by immediate return of circulation. Prior to the arrest, he had demonstrated lower limb movement. IONM did not demonstrate significant reductions or alerts. MRI was not performed due to the presence of a deep brain stimulator. CT showed no evidence of large intracranial or spinal bleeding, or malpositioned instrumentation. He spent limited time below a 60 mmHg threshold (normalised AUT 0.02 mmHg*min/min, normalised TUT 0.28%) and required vasoactive intervention; his minimum haemoglobin was 125 g/L, and he exhibited low urine output.

#### Cardiac and respiratory

One patient suffered myocardial damage evidenced by troponin rise requiring vasopressor support with central line insertion. Eight patients needed prolonged respiratory support: two due to sepsis, one due to respiratory failure, one due to spontaneous pneumothorax, and one due to aspiration. Three patients had significant aspiration events or lower respiratory tract infections.

#### Acute kidney injury

A total of 14 patients met UO criteria for Stage 1 AKI, with most occurrences in the second 12 h following PICU admission. Five patients met creatinine criteria for Stage 1 AKI, primarily between 8 and 20 h following PICU admission. No Stage 2 or 3 AKI occurred. Logistic regression of normalised AUT/TUT on AKI criteria showed no significant results.

## Discussion

This study reveals limited total hypotension exposure across the cohort below 60 and 65 mmHg MAP thresholds, as evidenced by the normalised AUT and TUT analysis. However, a significant proportion of patients (14.9%) experienced at least one episode of hypotension under the 60 mmHg threshold lasting ≥ 60 min, and 37.6% under the 65 mmHg threshold (Fig. 2). This suggests that some individuals can tolerate substantial exposure without apparent negative impact. Despite demographic uniformity, significant BP variability was observed (Fig. 3). When considering lactate as a proxy marker of tissue perfusion, our findings indicate that hypotension exposure below thresholds of 60 and 65 mmHg for periods ≥ 60 min was associated with lactate elevation. Close monitoring of urine output and lactate levels may help identify and protect at-risk patients. Optimising post-operative haemoglobin may lessen the compounding effect of hypotension exposure on lactate.

The ambiguity surrounding definitions for paediatric hypotension complicates blood pressure management in this population. There are several clinical aged-stratified guidelines to direct paediatric blood pressure management [19–21, 50]. A 2019 systematic review revealed a lack of agreement between clinical guidelines and population-based lower centiles. Numerous articles discussed paediatric intra-operative MAP targets during spinal surgery, proposing hypotension definitions of 60 and 65 mmHg, with authors reporting a reduction in IONM signals below 60 mmHg [9, 51–54]. However, most literature regarding peri-operative haemodynamic targets predominantly reported on cohorts with adolescent idiopathic scoliosis. Authors of a retrospective study defined a clinically significant hypotensive event as MAP < 60 mmHg. Patients who were hemodynamically stable in the first four hours after the surgery did not sustain a hypotensive event at any later point in their post-operative course, and patients who were hypotensive post-operatively were mainly hypotensive within the first four hours post-operatively [55]. A prospective study of 59 AIS patients found that intra-operative goal-directed haemodynamic therapy, with MAP targets > 60 mmHg (or > 75 mmHg with depressed MEPs), resulted in shorter durations of hypotension, truncated hospital stays, and a smaller reduction in haemoglobin post-operatively [53].

DSCI, while rare, remains a significant concern, with its aetiology often uncertain. Proposed mechanistic aetiologies of DSCI include bone graft migration, stretch-induced ischaemia, post-operative cord oedema, and spinal artery vasospasm [7, 10, 11, 15–18]. Impaired autoregulation post-operatively may result in some patients being more susceptible to hypotension [9]. However, many patients experience short periods of hypotension without identifiable consequence. Two patients in our study sustained permanent neurological deficits, one of whom experienced longer periods of hypotension below the 60 mmHg threshold while anaemic. Although our data demonstrates that periods of post-operative hypotension are common and tolerated in most patients, this case provides support for the theory that for some individual patients, impaired spinal cord perfusion and oxygen delivery may be important in the mechanism of DSCI.

Oxygen delivery to tissues is the product of blood flow and arterial oxygen content [56]. Anaemia reduces oxygen content by lowering haemoglobin concentration, while hypotension impairs tissue perfusion by reducing blood flow, particularly when perfusion pressure falls below the threshold for autoregulation. When both factors are present, their combined effect substantially compromises oxygen delivery, increasing the risk of tissue hypoxia and organ dysfunction.

Patients with Rett syndrome are known to have significant dysautonomia, which manifests as reduced heart rate variability, prolonged QTc intervals, and impaired cardiorespiratory coupling [57]. These autonomic disturbances contribute to the increased risk of sudden death observed in this population [58]. The underlying pathophysiology involves brainstem dysfunction affecting cardiorespiratory control, leading to electrical instability of the cardiovascular system and heightened susceptibility to arrhythmias.

While the cardiac arrest patient did not have Rett syndrome, the suspected dysautonomic aetiology underscores the broader clinical relevance of autonomic dysfunction across different paediatric neurological conditions. Both cases demonstrate how dysautonomia, whether associated with specific genetic syndromes or occurring as an isolated phenomenon, can significantly impact cardiovascular stability and influence clinical outcomes in the peri-operative period.

Episodes of brief, profound hypotension associated with cardiac arrest, such as observed in one SCI patient, are mechanistically distinct from sustained periods of hypoperfusion. While these events result in extreme but short-lived MAP reductions, they do not represent the same physiological process as prolonged hypotension.

MAP is a sensitive but poorly specific parameter and clinicians may consider the risks of over-intervention, whereby targeting higher BP thresholds may lead to increased vasopressor use and therefore their associated complications in children who might otherwise tolerate brief periods of hypotension. Given the rarity of DSCI, a data-driven approach to determine a definitive BP safety threshold with DSCI as a sole dependent outcome is not feasible, necessitating the study of proxy markers as indicators of tissue perfusion.

While the current study's primary strength is its homogeneous cohort, the small sample size limits generalisability. The limited representation of younger age groups, given the nature of NMS surgical intervention, combined with the small cohort size, makes developing age-stratified threshold targets challenging. Hypotension exposure below 60 mmHg for patients who received vasopressor therapy was significantly greater than the remainder of the cohort. This may suggest that treating physicians consider an adequate MAP for this population to be around 60 mmHg and not higher. Ward-based urine output data for the 24-h period following the initial 24-h monitoring period were not collected. The authors agree that this is a limitation of the study, whereby AKI diagnosis meeting urine output criteria may be underreported. However, given the rapidly responsive nature of urine output, low urine output observed following discharge is more likely to be reflective of more recent physiology.

This work provides a foundation for the improved understanding of BP management and neurological surveillance in the non-communicative or cognitively impaired post-operative neuromuscular population. Prospective studies with larger cohorts are needed to validate optimal MAP targets and evaluate clinical interventions aimed at maintaining adequate tissue perfusion in NMS patients following extensive spinal deformity correction surgery. Future research should aim to address the limitations identified in this study and further explore therapeutic physiologic targets and patient outcomes in this complex population.

## Supplementary Information

Below is the link to the electronic supplementary material.

Supplementary file1 (DOCX 49 KB)

## Data Availability

The datasets generated during and/or analysed during the current study are not publicly available, but are available from the corresponding author on reasonable request.
