# Supplementary material for: Paediatric neuromuscular scoliosis and post-operative blood pressure targets: a retrospective analysis
Source: Spine Deform. 2025 Oct 24;14(2):517–29. doi: 10.1007/s43390-025-01200-1 (PMC12909380; doi:10.1007/s43390-025-01200-1)
Supplement: Supplementary file 1 — Supplementary file1 (DOCX 49 KB) [file 43390_2025_1200_MOESM1_ESM.docx]

| **Outcome** | **Value** |
| --- | --- |
| **Hypotension** |  |
| Severity thresholds | MAP of 40-80 mmHg in 5 mmHg increments^19–24,26,27^ |
| Duration | Episodes lasting 5, 10, 30, 45, 60, 90, 120 minutes |
| Pre-measurement period | 30, 60, 120 minutes |
| **Lactate** |  |
| Binary threshold | ≥ 2 mmol/L^40^ |
| Continuous | 0 - ∞ |
| **Haemoglobin (Hb)** |  |
| Categorical | *≤80, 80-100 and >100 g/L* |
| Continuous | 0 - ∞ |
| **Urine Output** |  |
| Binary threshold | <0.5 ml/kg/hr ^38,45^ |
| Continuous | 0 - ∞ |
| **Acute Kidney Injury (AKI)** |  |
| Binary threshold  *(for each stage)* | UO and Creatinine Kidney Disease Improving Global Outcomes (KDIGO) Criteria ^38,45^. *Cr collected up to 72 hours from ICU admission. eGFR criteria not utilised as height data was not available for collection.* |

***Supplementary Table 1.*** *Statistical Analysis Outcome Criteria*

| **Statistic** | **Value** |
| --- | --- |
| Lactate Mean (95% CI) | 1.44 (1.36-1.51) |
| Lactate Median | 1.2 |
| Lactate SD | 1.00 |
| Lactate Range | 0.3 - 9.3 |
| Total number lactate of readings | 419 |
| Total patients with a lactate reading ≥ 2 mmol/L | 44 (n = 99) |
| Total patients with a lactate reading ≥ 4 mmol/L | 6 (n = 21) |
| Mean number of lactate readings per patient | 4.19 |
| Total patients with at least one lactate reading | 99 |
| Total patients with normalised lactate (<2 mmol/L) at 24hrs | 74 |
| Total patients With Hb ≤ 70 g/L | 10 |
| Hb Values ≤ 70 g/L | 14 |
| Total patients With Hb ≤ 80 g/L | 27 |
| Hb Values ≤ 80 g/L | 75 |
| Total patients who Received RBC Transfusion | 9 |
| Total patients With Hb <= 70 g/L Received RBC | 4 |
| Total patients With Hb <= 80 g/L Received RBC | 8 |

***Supplementary Table 2.*** *Post-Operative Lactate and Haemoglobin Readings: Descriptive Statistics*

| **Pre-Measurement Period** | **MAP Threshold** | **TUT β Estimate** | **TUT 95% CI** | **TUT SE** | **TUT OR** | **TUT *p*** |
| --- | --- | --- | --- | --- | --- | --- |
| **30** | 60 | 20.25 | -0.12 - 40.61 | 10.39 | 620 806 622 | 0.05 |
| **30** | 65 | 12.50 | -1.32 - 26.33 | 7.05 | 269 267 | 0.08 |
| **30** | 70 | 7.07 | -3.97 - 18.12 | 5.64 | 1181 | 0.21 |
| **30** | 75 | 3.62 | -7.14 - 14.38 | 5.49 | 37 | 0.51 |
| **30** | 80 | -3.66 | -15.44 - 8.12 | 6.01 | 0.03 | 0.54 |
| **60** | 55 | 23.96 | -4.94 - 52.86 | 14.75 | 25 378 501 406 | 0.10 |
| **60** | 60 | 17.68 | 4.96 - 30.41 | 6.49 | 47 790 843 | 0.006 |
| **60** | 65 | 8.41 | 0.54 - 16.28 | 4.02 | 4490 | 0.04 |
| **60** | 70 | 4.64 | -1.64 - 10.92 | 3.20 | 103 | 0.15 |
| **60** | 75 | 1.70 | -4.10 - 7.51 | 2.96 | 6 | 0.56 |
| **60** | 80 | -2.18 | -8.68 - 4.31 | 3.31 | 0.11 | 0.51 |
| **120** | 50 | 21.44 | -20.75 - 63.62 | 21.52 | 2 040 878 663 | 0.32 |
| **120** | 55 | 17.29 | -1.78 - 36.36 | 9.73 | 32 276 948 | 0.08 |
| **120** | 60 | 7.90 | 1.43 - 14.36 | 3.30 | 2688 | 0.02 |
| **120** | 65 | 4.11 | -0.19 - 8.42 | 2.20 | 61 | 0.06 |
| **120** | 70 | 3.43 | -0.21 - 7.07 | 1.86 | 31 | 0.07 |
| **120** | 75 | 2.54 | -1.06 - 6.14 | 1.84 | 13 | 0.17 |
| **120** | 80 | 1.56 | -2.59 - 5.72 | 2.12 | 5 | 0.46 |

***Supplementary Table 3.*** *Multivariable Logistic Regression Analysis of TUT effect (continuous) on lactate (dichotomous), with age controlled for as a covariate. TUT values rescaled to hours (from minutes). ORs indicates odds ratios; SE indicates standard error; CI indicates confidence intervals.*

| **Pre-Measurement Period** | **MAP Threshold** | **AUT β Estimate** | **AUT 95% CI** | **AUT SE** | **AUT OR** | **AUT p** |
| --- | --- | --- | --- | --- | --- | --- |
| 30 | 45 | -2.89 | -11.33 - 5.56 | 4.31 | 0.06 | 0.50 |
| 30 | 50 | 0.36 | -0.58 - 1.29 | 0.48 | 1.43 | 0.45 |
| 30 | 55 | 0.22 | -0.11 - 0.55 | 0.17 | 1.24 | 0.20 |
| 30 | 60 | 0.20 | 0.05 - 0.36 | 0.08 | 1.22 | 0.01 |
| 30 | 65 | 0.13 | 0.05 - 0.22 | 0.04 | 1.14 | 0.002 |
| 30 | 70 | 0.11 | 0.05 - 0.17 | 0.03 | 1.12 | <.001 |
| 30 | 75 | 0.09 | 0.05 - 0.13 | 0.02 | 1.10 | <.001 |
| 30 | 80 | 0.07 | 0.04 - 0.11 | 0.02 | 1.08 | <.001 |
| 60 | 45 | -1.68 | -6.35 - 3.00 | 2.39 | 0.19 | 0.48 |
| 60 | 50 | -0.02 | -0.54 - 0.50 | 0.27 | 0.98 | 0.94 |
| 60 | 55 | 0.06 | -0.12 - 0.25 | 0.09 | 1.06 | 0.51 |
| 60 | 60 | 0.08 | -0.01 - 0.16 | 0.04 | 1.08 | 0.08 |
| 60 | 65 | 0.06 | 0.01 - 0.10 | 0.02 | 1.06 | 0.02 |
| 60 | 70 | 0.05 | 0.02 - 0.08 | 0.02 | 1.05 | 0.002 |
| 60 | 75 | 0.05 | 0.02 - 0.07 | 0.01 | 1.05 | <.001 |
| 60 | 80 | 0.04 | 0.02 - 0.06 | 0.01 | 1.04 | <.001 |
| 120 | 45 | -2.10 | -6.16 - 1.95 | 2.07 | 0.12 | 0.31 |
| 120 | 50 | -0.14 | -0.52 - 0.24 | 0.19 | 0.87 | 0.47 |
| 120 | 55 | 0.00 | -0.11 - 0.12 | 0.06 | 1.00 | 0.96 |
| 120 | 60 | 0.03 | -0.02 - 0.08 | 0.03 | 1.03 | 0.24 |
| 120 | 65 | 0.03 | -0.00 - 0.05 | 0.01 | 1.03 | 0.07 |
| 120 | 70 | 0.02 | 0.01 - 0.04 | 0.01 | 1.02 | 0.01 |
| 120 | 75 | 0.02 | 0.01 - 0.03 | 0.01 | 1.02 | 0.003 |
| 120 | 80 | 0.02 | 0.01 - 0.03 | 0.01 | 1.02 | 0.002 |

***Supplementary Table 4****. Multivariable Logistic Regression Analysis of AUT effect (continuous) on UO (dichotomous). AUT values rescaled to mmHg*hours (from mmHg*mins). ORs indicates odds ratios; SE indicates standard error; CI indicates confidence intervals.*

| **Pre-Measurement Period** | **MAP Threshold** | **TUT β Estimate** | **TUT 95% CI** | **TUT SE** | **TUT OR** | **TUT *p*** |
| --- | --- | --- | --- | --- | --- | --- |
| 30 | 45 | -5.14 | -22.17 - 11.88 | 8.68 | 0.01 | 0.55 |
| 30 | 50 | 2.10 | -1.54 - 5.74 | 1.86 | 8.17 | 0.26 |
| 30 | 55 | 1.57 | -0.28 - 3.41 | 0.94 | 4.79 | 0.10 |
| 30 | 60 | 1.55 | 0.56 - 2.54 | 0.51 | 4.71 | 0.002 |
| 30 | 65 | 1.13 | 0.39 - 1.86 | 0.37 | 3.08 | 0.003 |
| 30 | 70 | 1.40 | 0.75 - 2.06 | 0.33 | 4.07 | <.001 |
| 30 | 75 | 1.34 | 0.69 - 1.99 | 0.33 | 3.81 | <.001 |
| 30 | 80 | 1.22 | 0.49 - 1.95 | 0.37 | 3.38 | 0.001 |
| 60 | 45 | -3.27 | -12.94 - 6.39 | 4.93 | 0.04 | 0.51 |
| 60 | 50 | 0.57 | -1.51 - 2.64 | 1.06 | 1.76 | 0.59 |
| 60 | 55 | 0.66 | -0.40 - 1.72 | 0.54 | 1.94 | 0.22 |
| 60 | 60 | 0.72 | 0.17 - 1.27 | 0.28 | 2.06 | 0.01 |
| 60 | 65 | 0.59 | 0.20 - 0.99 | 0.20 | 1.81 | 0.003 |
| 60 | 70 | 0.78 | 0.43 - 1.13 | 0.18 | 2.18 | <.001 |
| 60 | 75 | 0.78 | 0.43 - 1.13 | 0.18 | 2.18 | <.001 |
| 60 | 80 | 0.67 | 0.28 - 1.06 | 0.20 | 1.95 | <.001 |
| 120 | 45 | -3.73 | -11.54 - 4.08 | 3.98 | 0.02 | 0.35 |
| 120 | 50 | -0.13 | -1.46 - 1.20 | 0.68 | 0.88 | 0.85 |
| 120 | 55 | 0.26 | -0.38 - 0.89 | 0.33 | 1.29 | 0.43 |
| 120 | 60 | 0.32 | 0.00 - 0.63 | 0.16 | 1.38 | 0.05 |
| 120 | 65 | 0.30 | 0.08 - 0.52 | 0.11 | 1.35 | 0.008 |
| 120 | 70 | 0.36 | 0.16 - 0.55 | 0.10 | 1.43 | <.001 |
| 120 | 75 | 0.34 | 0.15 - 0.53 | 0.10 | 1.40 | <.001 |
| 120 | 80 | 0.31 | 0.10 - 0.52 | 0.11 | 1.36 | 0.004 |

***Supplementary Table 5.*** *Multivariable Logistic Regression Analysis of TUT effect (continuous) on UO (dichotomous). TUT values rescaled to hours (from minutes). ORs indicates odds ratios; SE indicates standard error; CI indicates confidence intervals.*

| **Pre-Measurement Period** | **MAP Threshold** | **Hb ≤80 g/L β Estimate** | **Hb ≤80 g/L 95% CI** | **Hb ≤80 g/L SE** | **Hb ≤80 g/L OR** | **Hb ≤80 g/L *p*** |
| --- | --- | --- | --- | --- | --- | --- |
| 30 | 55 | 1.44 | -0.21 - 3.09 | 0.84 | 4.21 | 0.09 |
| 30 | 60 | 1.65 | -0.02 - 3.32 | 0.85 | 5.20 | 0.05 |
| 30 | 65 | 1.73 | 0.03 - 3.43 | 0.87 | 5.63 | 0.046 |
| 30 | 70 | 1.52 | -0.13 - 3.16 | 0.84 | 4.57 | 0.07 |
| 30 | 75 | 1.53 | -0.12 - 3.18 | 0.84 | 4.62 | 0.07 |
| 30 | 80 | 1.82 | 0.00 - 3.64 | 0.93 | 6.18 | 0.05 |
| 60 | 60 | 1.50 | -0.27 - 3.26 | 0.90 | 4.47 | 0.10 |
| 60 | 65 | 1.54 | -0.20 - 3.28 | 0.89 | 4.67 | 0.08 |
| 120 | 50 | 1.86 | -0.65 - 4.38 | 1.28 | 6.45 | 0.15 |
| 120 | 55 | 1.65 | -0.62 - 3.92 | 1.16 | 5.22 | 0.15 |
| 120 | 60 | 1.55 | -0.64 - 3.75 | 1.12 | 4.72 | 0.17 |
| 120 | 65 | 1.60 | -0.61 - 3.82 | 1.13 | 4.97 | 0.16 |
| 120 | 70 | 1.58 | -0.73 - 3.90 | 1.18 | 4.87 | 0.18 |
| 120 | 75 | 1.68 | -0.71 - 4.07 | 1.22 | 5.37 | 0.17 |
| 120 | 80 | 1.98 | -0.53 - 4.48 | 1.28 | 7.21 | 0.12 |

***Supplementary Table 6.*** *Multivariable Logistic Regression Analysis of haemoglobin (categorical ≤80 g/L against >100 g/), with TUT (continuous) on lactate (dichotomous) and age controlled for as a covariate. TUT values rescaled to hours (from minutes). ORs indicates odds ratios; SE indicates standard error; CI indicates confidence intervals.*
